# Supplementary material for: A miRNA-based diagnostic model predicts resectable lung cancer in humans with high accuracy
Source: Commun Biol. 2020 Mar 19;3:134. doi: 10.1038/s42003-020-0863-y (PMC7081195; doi:10.1038/s42003-020-0863-y)
Supplement: Supplementary file 1 — Supplementary Information [file 42003_2020_863_MOESM1_ESM.pdf]

Supplementary Table 1. Top 20 miRNAs in the discovery set

microRNAs	AUC	Cross-validation score
miR-17-3p	0.9346	0.9087
miR-6877-5p	0.9491	0.8822
miR-1343-3p	0.969	0.899
miR-187-5p	0.9126	0.8702
miR-1268b	0.9374	0.8678
miR-4787-3p	0.9352	0.8606
miR-6765-5p	0.9254	0.8534
miR-1199-5p	0.9206	0.8726
miR-6075	0.8913	0.8269
miR-4258	0.8983	0.8317
miR-668-5p	0.8934	0.8365
miR-8073	0.9058	0.8341
miR-6511b	0.8943	0.8269
miR-320b	0.8868	0.8221
miR-4417	0.8815	0.8173
miR-6789-5p	0.8925	0.8245
miR-8060	0.8779	0.8173
miR-1228-5p	0.9175	0.8486
miR-4740-5p	0.8884	0.8173
miR-29b-3p	0.8607	0.8149

Supplementary Table2. Top 20 microRNA combinations in model2 (discovery set)

microRNAs	AUC	Cross-validation score
miR-1268b + miR-6075	0.9934	0.9904
miR-6765-5p + miR-6836-3p	0.9932	0.9736
miR-6765-5p + miR-4634	0.992	0.9736
miR-187-5p + miR-6765-5p	0.9942	0.976
miR-1343-3p + miR-4632-5p	0.993	0.9736
miR-1343-3p + miR-4728-5p	0.9947	0.9736
miR-1343-3p + miR-197-5p	0.9942	0.9736
miR-1343-3p + miR-6887-5p	0.9912	0.9736
miR-1343-3p + miR-4419b	0.9946	0.9808
miR-1268b + miR-7108-5p	0.9875	0.9688
miR-1343-3p + miR-3619-3p	0.9923	0.9639
miR-1268b + miR-4467	0.9813	0.9688
miR-1343-3p + miR-6858-5p	0.9939	0.9639
miR-6765-5p + miR-6789-5p	0.9895	0.9663
miR-6877-5p + miR-4632-5p	0.9901	0.9591
miR-187-5p + miR-1228-5p	0.9925	0.9663
miR-187-5p + -miR-1268b	0.9826	0.9519
miR-6877-5p + miR-1268b	0.9915	0.9567
miR-1343-3p + miR-6787-5p	0.9852	0.9663
miR-1268b + miR-6836-3p	0.9864	0.9519

Supplementary Table3. Univariable and age-adjusted logistic regression analyses

	univariable<br>OR (95% CI)	adjusted<br>OR (95% CI)
Two-miRNA panel<br>(MiR-1268b + miR-6075)	21.76 (15.98–29.63)	20.34 (13.99 - 29.57)
miR-1268b	12.60 (10.60-14.97)	11.03 (9.00 - 13.52)
miR-6075	7.10 (6.24-8.07)	7.48 (6.32 - 8.85)

Data are expressed as the odds ratio (95% confidence interval).

Supplementary Table 4. Top 100 microRNAs in serum samples from NCCH and YMC (* indicates three internal control microRNAs)

microRNAs	NCCH	YMC
*hsa-miR-149-3p	1	40
hsa-miR-4763-3p	2	63
hsa-miR-6821-5p	3	52
hsa-miR-1268b	4	12
hsa-miR-6756-5p	5	64
hsa-miR-4632-5p	6	60
hsa-miR-6858-5p	7	65
hsa-miR-4687-3p	8	36
hsa-miR-4689	9	77
hsa-miR-4530	10	99
hsa-miR-6879-5p	11	93
hsa-miR-6085	12	54
hsa-miR-6791-5p	13	51
hsa-miR-4459	14	>100
hsa-miR-6802-5p	15	61
hsa-miR-937-5p	16	74
hsa-miR-5001-5p	17	87
hsa-miR-4695-5p	18	68
hsa-miR-6785-5p	19	97
hsa-miR-1343-5p	20	42
hsa-miR-7108-5p	21	47
hsa-miR-6798-5p	22	43
hsa-miR-4442	23	66
hsa-miR-3197	24	67
hsa-miR-6781-5p	25	13
hsa-miR-6743-5p	26	80
hsa-miR-6765-5p	27	25
hsa-miR-6816-5p	28	18
hsa-miR-4758-5p	29	55
*hsa-miR-4463	30	45
hsa-miR-1268a	31	11
hsa-miR-6803-5p	32	6

hsa-miR-4665-5p	33	82
hsa-miR-4505	34	83
hsa-miR-4294	35	>100
hsa-miR-4706	36	81
hsa-miR-4723-5p	37	96
hsa-miR-6757-5p	38	>100
hsa-miR-92b-5p	39	70
hsa-miR-1227-5p	40	30
hsa-miR-6768-5p	41	88
hsa-miR-6724-5p	42	73
hsa-miR-6845-5p	43	58
hsa-miR-6780b-5p	44	92
hsa-miR-6752-5p	45	16
hsa-miR-328-5p	46	34
hsa-miR-4467	47	95
hsa-miR-1228-5p	48	35
hsa-miR-4739	49	57
hsa-miR-6749-5p	50	49
hsa-miR-3656	51	17
hsa-miR-6726-5p	52	>100
hsa-miR-4651	53	33
hsa-miR-6805-5p	54	32
hsa-miR-6789-5p	55	41
hsa-miR-4492	56	53
hsa-miR-8072	57	39
hsa-miR-6126	58	86
hsa-miR-6125	59	8
hsa-miR-1908-5p	60	27
hsa-miR-6786-5p	61	2
*hsa-miR-2861	62	19
hsa-miR-6850-5p	63	20
hsa-miR-1915-3p	64	84
hsa-miR-3196	65	44
hsa-miR-3940-5p	66	22
hsa-miR-4484	67	75

hsa-miR-1237-5p	68	46
hsa-miR-4466	69	14
hsa-miR-8069	70	9
hsa-miR-6089	71	3
hsa-miR-4508	72	15
hsa-miR-4488	73	5
hsa-miR-6784-5p	74	21
hsa-miR-6090	75	1
hsa-miR-4281	76	62
hsa-miR-638	77	7
hsa-miR-6729-5p	78	4
hsa-miR-3178	79	31
hsa-miR-3619-3p	80	98
hsa-miR-1260b	81	>100
hsa-miR-663a	82	71
hsa-miR-1469	83	59
hsa-miR-642b-3p	84	94
hsa-miR-6787-5p	85	56
hsa-miR-7977	86	>100
hsa-miR-7975	87	>100
hsa-miR-5787	88	38
hsa-miR-3621	89	50
hsa-miR-3960	90	23
hsa-miR-4787-5p	91	24
hsa-miR-6087	92	69
hsa-miR-6727-5p	93	28
hsa-miR-1343-3p	94	>100
hsa-miR-4649-5p	95	85
hsa-miR-6088	96	89
hsa-miR-4258	97	>100
hsa-miR-762	98	37
hsa-miR-128-2-5p	99	91
hsa-miR-7704	100	29

Supplementary Methods

Algorithm: combinatorial optimization for multicandidate miRNAs

Notation:

Nm: Number of miRNAs

mR(i): The ith miRNA where i = 1 TO Nm

Ns: Number of miRNA sets with better performance to limit at each step.

Mc: Maximum number of miRNA combinations you want to examine.

Sc: Score (same as Accuracy) = (TP + TN) / (TP + FP + FN + TN), where the meaning of each variable is as follows:

	cancer	no cancer
prediction positive	TP: number of true positives	FP: number of false positives
prediction negative	FN: number of false negatives	TN: number of true negatives

LDA-LOO(): Function of Linear Discriminant Algorithm (LDA) with Leave One Out Cross Validation (LOO-CV). We used LDA function included as lda() in R language MASS package version 7.3-45. The score (Sc) by LOO-CV is calculated as accumulated number, counting up the results (hit or not) of evaluating each leave-out sample, divided by number of trials.

Processing steps:

Step 1: Divide the data to be analyzed into halves for training data and test data.

Step 2: Set Ns=20 and Mc=10.

(For training data)

Step 3: Combinatorial optimization

```
        FOR I = 1 TO Nm
                Sc[i] = LDA-LOO( mR(i) )
```

```
miRNA_index_of_sorted[1:Nm] = ORDER( Sc[1:Nm], descend order )
Pivot[1:Ns] = miRNA_index_of_sorted[1:Ns]     # get indexes of top Ns as Pivot
i = 1
FOR j = 1 TO Mc
        Sc = 0
        FOR k = 1 TO Nm
                IF k is not included in Pivot_set[j] {Sc[i] =
                LDA-LOO( mR[Pivot_set[j]], mR[k] )}
                i = i + 1
        miRNA_set[Ns * (Nm-i)] = ORDER( Sc[Ns * (Nm-i)], descend order )
        Pivot_set[1:Ns] = miRNA_set[1:Ns]     # get indexes of top Ns as Pivot
```

Step 4: Calculate coefficients of Ns set of linear discriminant models.

Step 5: Calculate INDEX: An offset is added to the discrimination model so that the cutoff value becomes 0.

Step 6: Calculate performances for Ns discriminant models with sensitivity, specificity, accuracy, positive predictive value, negative predictive value, area under the ROC curve on the training data.

(For test data)

Step 7: calculate upper same performances using Ns discriminant models for test data.
