# Supplementary material for: A miRNA-based diagnostic model predicts resectable lung cancer in humans with high accuracy
Source: Commun Biol. 2020 Mar 19;3:134. doi: 10.1038/s42003-020-0863-y (PMC7081195; doi:10.1038/s42003-020-0863-y)
Supplement: Supplementary file 2 — Description of Additional Supplementary Items [file 42003_2020_863_MOESM2_ESM.pdf]

Description of additional supplementary items

Supplementary Data 1. Source data of Figure 2b, c
Supplementary Data 2. Source data of Figure 3a
Supplementary Data 3. Source data of Figure 3b, c
Supplementary Data 4. Source data of Figure 4
Supplementary Data 5. Source data of Figure 5
Supplementary Data 6. Source data of Figure 6
Supplementary Data 7. R script used for combinational optimization for multicandidate miRNAs (R file)
