# Supplementary material for: A miRNA-based diagnostic model predicts resectable lung cancer in humans with high accuracy
Source: Commun Biol. 2020 Mar 19;3:134. doi: 10.1038/s42003-020-0863-y (PMC7081195; doi:10.1038/s42003-020-0863-y)
Supplement: Supplementary file 10 — Reporting Summary [file 42003_2020_863_MOESM10_ESM.pdf]

nature research

Corresponding author(s): Ochiya Takahiro

Last updated by author(s): Dec 3, 2019

Reporting Summary

Nature Research wishes to improve the reproducibility of the work that we publish. This form provides structure for consistency and transparency in reporting. For further information on Nature Research policies, see Authors & Referees and the Editorial Policy Checklist.

Statistics

For all statistical analyses, confirm that the following items are present in the figure legend, table legend, main text, or Methods section.

n/a	Confirmed	
☐	☒	The exact sample size (*n*) for each experimental group/condition, given as a discrete number and unit of measurement
☐	☒	A statement on whether measurements were taken from distinct samples or whether the same sample was measured repeatedly
☐	☒	The statistical test(s) used AND whether they are one- or two-sided<br>*Only common tests should be described solely by name; describe more complex techniques in the Methods section.*
☐	☒	A description of all covariates tested
☐	☒	A description of any assumptions or corrections, such as tests of normality and adjustment for multiple comparisons
☐	☒	A full description of the statistical parameters including central tendency (e.g. means) or other basic estimates (e.g. regression coefficient) AND variation (e.g. standard deviation) or associated estimates of uncertainty (e.g. confidence intervals)
☐	☒	For null hypothesis testing, the test statistic (e.g. *F*, *t*, *r*) with confidence intervals, effect sizes, degrees of freedom and *P* value noted<br>*Give P values as exact values whenever suitable.*
☒	☐	For Bayesian analysis, information on the choice of priors and Markov chain Monte Carlo settings
☐	☒	For hierarchical and complex designs, identification of the appropriate level for tests and full reporting of outcomes
☐	☒	Estimates of effect sizes (e.g. Cohen's *d*, Pearson's *r*), indicating how they were calculated

Our web collection on statistics for biologists contains articles on many of the points above.

Software and code

Policy information about availability of computer code

Data collection	No software was used for data collection.
Data analysis	Statistical analyses were performed using R version 3.1.2 (R Foundation for Statistical Computing, http://www.R-project.org), compute.es version 0.2-4, hash version 2..26, MASS version 7.3-45, mutoss version 0.1-10, pROC version 1.8, SPSS version 25 (IBM Corp, Armonk, NY), and GraphPad Prism version 7 (GraphPad Software, La Jolla, CA). Unsupervised clustering and heat map generation using Pearson's correlation in Ward's method for linkage analysis, as well as principal component analysis (PCA), were performed using Partek Genomics Suite 6.6.

For manuscripts utilizing custom algorithms or software that are central to the research but not yet described in published literature, software must be made available to editors/reviewers. We strongly encourage code deposition in a community repository (e.g. GitHub). See the Nature Research guidelines for submitting code & software for further information.

Data

Policy information about availability of data

All manuscripts must include a data availability statement. This statement should provide the following information, where applicable:

- Accession codes, unique identifiers, or web links for publicly available datasets
- A list of figures that have associated raw data
- A description of any restrictions on data availability

All miRNA microarray data and clinical information on the patients who provided the serum samples will be deposited in the Gene Expression Omnibus (GEO) (https://www.ncbi.nlm.nih.gov/geo/) database before the acceptance. All other relevant data are available within the article file or Supplementary Information, or available from the authors on reasonable request.

Field-specific reporting

Please select the one below that is the best fit for your research. If you are not sure, read the appropriate sections before making your selection.

☒ Life sciences ☐ Behavioural & social sciences ☐ Ecological, evolutionary & environmental sciences

For a reference copy of the document with all sections, see nature.com/documents/nr-reporting-summary-flat.pdf

Life sciences study design

All studies must disclose on these points even when the disclosure is negative.

Sample size	Serial serum samples were collected preoperatively from lung cancer patients who underwent surgical resection at the National Cancer Center Hospital (NCCH)(n=1698). These samples were registered in the National Cancer Center (NCC) Biobank between 2008 and 2016. Non-cancer serum samples were collected from the Yokohama Minoru Clinic (YMC) (n=1998) and from patients who were not diagnosed with any cancer based on the results of imaging examination or biopsy at NCCH (n=207).Because the samples were chronologically divided into two cohorts, the sample size for each cohort could not be determined based on a pre-specified effect size.
Data exclusions	Among the 1698 lung cancer serum samples, 74 were excluded due to low-quality microarray results, 33 due to past history of other cancers, 25 due to lack of patient information, 19 due to treatment before collection of serum, and 4 because the interval between serum collection and surgery was greater than 180 days, leaving 1566 samples for analysis.
Replication	The reproducibility of the microarray analysis was confirmed by performing microarray analysis on the same RNA sample for fifteen times. A strong correlation between the fifteen replicates was indicated (Pearson's correlation coefficient [R], 0.96 [95% confidence interval, 0.94-0.98])
Randomization	The training and validation cohorts were randomly divided using computer-generated random numbers.
Blinding	The collection of clinical information and miRNA expression analysis were blindly performed by different members.

Reporting for specific materials, systems and methods

We require information from authors about some types of materials, experimental systems and methods used in many studies. Here, indicate whether each material, system or method listed is relevant to your study. If you are not sure if a list item applies to your research, read the appropriate section before selecting a response.

Materials & experimental systems

n/a	Involved in the study	
☒	☐	Antibodies
☒	☐	Eukaryotic cell lines
☒	☐	Palaeontology
☒	☐	Animals and other organisms
☐	☒	Human research participants
☒	☐	Clinical data

Methods

n/a	Involved in the study	
☒	☐	ChIP-seq
☒	☐	Flow cytometry
☒	☐	MRI-based neuroimaging

Human research participants

Policy information about studies involving human research participants

Population characteristics	Lung cancer, NCC non-cancer, and YMC non-cancer samples were grouped into discovery and validation sets. The discovery set included 208 lung cancer, 104 NCC non-cancer, and 104 YMC non-cancer samples. The validation sets included 1358 lung cancer, 103 NCC non-cancer, and 1867 YMC non-cancer samples. Patient characteristics for the discovery and validation sets are shown in Table 1. In the discovery set, we observed no significant difference in patient characteristics, including age, sex, or smoking history, between the 208 lung cancer patients and 208 non-cancer participants. In the validation set, age was significantly higher in the 1358 lung cancer patients than in the 1970 non-cancer participants (66.3 ± 0.3 vs. 50.3 ± 0.2, p<0.001). In the validation set, the proportions of men and smokers were also significantly higher among lung cancer patients than among non-cancer participants (58% vs. 33%, p=0.002; 65% vs. 13%, p<0.0001, respectively).
Recruitment	Serial serum samples were collected preoperatively from lung cancer patients who underwent surgical resection at the National Cancer Center Hospital (NCCH)(n=1698). These samples were registered in the National Cancer Center (NCC) Biobank between 2008 and 2016. Non-cancer serum samples were collected from the Yokohama Minoru Clinic (YMC) (n=1998) and from patients who were not diagnosed with any cancer based on the results of imaging examination or biopsy at NCCH (n=207).
Ethics oversight	The study was approved by the NCCH Institutional Review Board (2015-376, 2016-249) and the Research Committee of Medical

Ethics oversight

Corporation Shintokai Yokohama Minoru Clinic (6019-18-3772). Written informed consent was obtained from each participant for their serum samples.

Note that full information on the approval of the study protocol must also be provided in the manuscript.
